# Effects of β-alanine supplementation and high-intensity interval training on endurance performance and body composition in men; a double-blind trial

**DOI:** 10.1186/1550-2783-6-5

**Published:** 2009-02-11

**Authors:** Abbie E Smith, Ashley A Walter, Jennifer L Graef, Kristina L Kendall, Jordan R Moon, Christopher M Lockwood, David H Fukuda, Travis W Beck, Joel T Cramer, Jeffrey R Stout

**Affiliations:** 1 Metabolic and Body Composition Laboratory, Department of Health and Exercise Science, University of Oklahoma, Norman, OK 73019, USA; 2 Biophysics Laboratory; Department of Health and Exercise Science, University of Oklahoma, Norman, OK 73019, USA

## Abstract

**Background:**

Intermittent bouts of high-intensity exercise result in diminished stores of energy substrates, followed by an accumulation of metabolites, promoting chronic physiological adaptations. In addition, β-alanine has been accepted has an effective physiological hydrogen ion (H^+^) buffer. Concurrent high-intensity interval training (HIIT) and β-alanine supplementation may result in greater adaptations than HIIT alone. The purpose of the current study was to evaluate the effects of combining β-alanine supplementation with high-intensity interval training (HIIT) on endurance performance and aerobic metabolism in recreationally active college-aged men.

**Methods:**

Forty-six men (Age: 22.2 ± 2.7 yrs; Ht: 178.1 ± 7.4 cm; Wt: 78.7 ± 11.9; VO_2_peak: 3.3 ± 0.59 l·min^-1^) were assessed for peak O_2 _utilization (VO_2_peak), time to fatigue (VO_2TTE_), ventilatory threshold (VT), and total work done at 110% of pre-training VO_2_peak (TWD). In a double-blind fashion, all subjects were randomly assigned into one either a placebo (PL – 16.5 g dextrose powder per packet; n = 18) or β-alanine (BA – 1.5 g β-alanine plus 15 g dextrose powder per packet; n = 18) group. All subjects supplemented four times per day (total of 6 g/day) for the first 21-days, followed by two times per day (3 g/day) for the subsequent 21 days, and engaged in a total of six weeks of HIIT training consisting of 5–6 bouts of a 2:1 minute cycling work to rest ratio.

**Results:**

Significant improvements in VO_2_peak, VO_2TTE_, and TWD after three weeks of training were displayed (p < 0.05). Increases in VO_2_peak, VO_2TTE_, TWD and lean body mass were only significant for the BA group after the second three weeks of training.

**Conclusion:**

The use of HIIT to induce significant aerobic improvements is effective and efficient. Chronic BA supplementation may further enhance HIIT, improving endurance performance and lean body mass.

## Background

High-intensity exercise results in diminished stores of adenosine tri-phosphate (ATP), phosphocreatine (PCr) and glycogenic substrates, and the intracellular accumulation of metabolites (adenosine di-phosphate (ADP), inorganic phosphate (P_i_), hydrogen ions (H^+^) and magnesium (Mg^+^), each of which has been implicated as a cause of muscle fatigue [1-3]. Excessive formation of H^+ ^results in a decrease in intramuscular pH which may contribute to fatigue in some models of exercise [1,4-6]. Enhancing an individual's ability to buffer protons may delay fatigue by improving the use of energy substrates and maintaining muscular contraction [6-9]. When the time and intensity level of exercise is sufficient, the majority of protons that are produced are buffered by the bicarbonate (HCO_3_^-^) buffering system [10,11] in which they are exported from the muscle [12]. Physiological buffering during dynamic exercise is typically controlled by the HCO_3_^- ^system and is also supported by direct physico-chemical buffering, provided mainly by phosphate, hisitidine residues of peptides and proteins, and the small amount of bicarbonate present in muscle at the start of exercise. However, during short bursts of intense exercise, such as HIIT, physico-chemical buffering will exceed that by HCO_3_^- ^mediated dynamic buffering, calling on intramuscular stores of phosphates and peptides.

Specifically, carnosine (β-alanyl-L-histidine), a cytoplasmic dipeptide, constitutes an important non-bicarbonate physico-chemical buffer. By virtue of a pKa of 6.83 and its high concentration in muscle, carnosine is more effective at sequestering protons than either bicarbonate (pKa 6.37) or inorganic phosphate (pKa 7.2), the other two major physico-chemical buffers over the physiological pH range [7,13]. However, as a result of the greater concentration of carnosine in muscle than bicarbonate in the initial stages of muscle contraction, and inorganic phosphate, its buffering contribution may be quantitatively more important.

Mechanisms for increasing muscle carnosine concentration have been somewhat disputed. While carnosine may be increased in chronically trained athletes, the effects of acute training are less clear. In one study, it has been reported that eight weeks of intensive training may increase intramuscular carnosine content [14]. In contrast, several other studies have shown that intense training, of up to 16 weeks, has been unable to promote a rise in skeletal muscle carnosine levels [6,15-17]. Only when β-alanine supplementation was combined with training did an increase in muscle carnosine occur [16], although the increase (40–60%) was similar to that seen with supplementation alone [18].

While carnosine is synthesized in the muscle from its two constituents, β-alanine and histidine [19], synthesis is limited by the availability of β-alanine [18,20]. β-alanine supplementation alone has been shown to significantly increase the intramuscular carnosine content [6,18]. Elevation of intramuscular carnosine content via β-alanine supplementation alone, has been shown to improve performance [6,14,21-24]. Recently, Hill and colleagues [6] demonstrated a 13% improvement in total work done (TWD) following four weeks of β-alanine supplementation, and an additional 3.2% increase after 10 weeks. Zoeller et al. [24] also reported significant increases in ventilatory threshold (VT) in a sample of untrained men after supplementing with β-alanine (3.2 g·d^-1^) for 28 days. In agreement, Kim et al. [21] also reported significant increases in VT and time to exhaustion (TTE) in highly trained male cyclists after 12 weeks of β-alanine (4.8 g·d^-1^) supplementation and endurance training. Furthermore, Stout et al. [22,23] reported a significant delay in neuromuscular fatigue, measured by physical working capacity at the fatigue threshold (PWC_FT_), in both men and women after 28 days of β-alanine supplementation (3.2 g·d^-1 ^– 6.4 g·d^-1^). Despite the improvements in VT, TTE, TWD, and PWC_FT _after supplementation, there were no increases in aerobic power, measured by VO_2_peak [22-24].

Although HIIT alone does not appear to increase skeletal muscle carnosine content [17], training has been suggested to improve muscle buffering capacity [25-27]. When repeated bouts of high-intensity intervals are interspersed with short rest periods, subsequent trials are initiated at a much lower pH [28]. Training in such a manner subjects the body to an acidic environment, forcing several physiological adaptations. Notably, HIIT has been shown to improve VO_2_peak and whole body fat oxidation in only two weeks (7 sessions at 90% VO_2_peak) [29]. Furthermore, over a longer period of time (4–6 weeks), HIIT has been reported to increase high-intensity exercise performance (6–21%), muscle buffering capacity, whole body exercise fat oxidation, and aerobic power (VO_2_peak) [25-27].

The respective supporting bodies of literature for the use of β-alanine supplementation alone and high-intensity training alone have gained recent popularity. However, to date, no study has combined and evaluated concurrent HIIT with β-alanine supplementation. In theory, we hypothesize that an increase in intramuscular carnosine content, as a result of β-alanine supplementation, may enhance the quality of HIIT by reducing the accumulation of hydrogen ions, leading to greater physiological adaptations. Therefore, the purpose of this study was to determine the effects of chronic (6 weeks) β-alanine supplementation in combination with HIIT on endurance performance measures in recreationally trained individuals.

## Methods

### Subjects

Forty-six college-aged men, who were recreationally active one to five hours per week, and had not taken any sports supplement within the six months prior-, volunteered to participate in this study (mean ± SD; Age: 22.2 ± 2.7 yrs, Height: 178.1 ± 7.4 cm, Weight: 78.7 ± 11.9 kg). Subjects were informed of the potential risks, benefits, and time requirements prior to enrolling and giving written consent. All study procedures were approved by the University's Institutional Review Board.

### Study design

This double-blind, randomized study included two three-week periods of HIIT and β-alanine supplementation. All participants completed a series of baseline, mid- and post-testing, including a series of cycling tests and body composition assessment using air displacement plethysmography (BodPod^®^) at all time points. Following baseline testing subjects were randomly assigned, in a double-blind fashion, to one of two supplementing groups, β-alanine or placebo, both with HIIT. Participant's initial VO_2_peak power output values were used to establish the TWD intensity and the training intensity for the six week duration, with no modification to intensity following mid-testing. The first three-week period of training was completed at workloads between 90%–110% of each individual's VO_2_peak, while the second three-week training peaked at 115%. While training, participants supplemented with 6 g per day of β-alanine or placebo during the first three weeks and 3 g per day during the second three week phase. Supplementing with 6.4 g per day of β-alanine, for 28 days has demonstrated a 60% increase in carnosine concentration [6,18], supporting the 21 day phase, allowing for an adequate loading period for β-alanine to elicit increases in intramuscular carnosine concentration. Furthermore, recent literature suggests even greater increases in carnosine levels when combining high-intensity training and β-alanine supplementation [17]. Following the three-week adaptation phase, mid-training and post-training tests were completed in the same order as the pre-testing, allowing at least 48 hours between each testing session. All subjects were instructed to maintain their current diet throughout the duration of the study and were asked to refrain from caffeine and vigorous activity 24 hours prior to any testing session. Food logs were distributed to all participants and completed (two non-consecutive weekdays and one weekend day) at baseline-testing, mid-testing and post-testing, to evaluate any changes in total kcal and/or protein intake.

### Determination of VO_2_peak

At pre-, mid-, and post-training, all participants performed a continuous graded exercise test (GXT) on an electronically braked cycle ergometer (Corval 400, Goningen, The Netherlands) to determine VO_2_peak, time to exhaustion (VO_2TTE_) and ventilatory threshold (VT). Pedal cadence was maintained at 70 rpm, while the power output was initially set at 50 W for a five minute warm-up, and increased by 25 W every two minutes, until the participant could no longer maintain the required power output (cadence dropped below 60 rpm). Respiratory gases were monitored breath by breath and analyzed with open-circuit spirometry (True One 2400^® ^Metabolic Measurement System, Parvo-Medics Inc., Provo UT) to determine VO_2_peak and VT. The data was averaged over 15 second intervals. The highest 15 second VO_2 _value during the GXT was recorded as the VO_2_peak value if it coincided with at least two of the following criteria: (a) a plateau in heart rate (HR) or HR values within 10% of the age-predicted HRmax, (b) a plateau in VO_2 _(defined by an increase of note more than 150 ml·min^-1^), and/or (c) an RER value greater than 1.15 [30]. Heart rate was also monitored continuously during exercise by using a heart rate monitor (Polar FS1, Polar Electro Inc. Lake Success, NY). The amount of time to reach exhaustion (VO_2TTE_) during the VO_2_peak was also recorded in seconds. Ventilatory threshold (VT) was determined using standard software (True One 2400^® ^Metabolic Measurement System, Parvo-Medics Inc., Provo UT) by plotting ventilation (V_E_) against VO_2 _as described previously [31]. Two linear regression lines were fit to the lower and upper portions of the V_E _vs. VO_2 _curve, before and after the break points, respectively. The intersection of these two lines was defined as VT, and was recorded with respect to the corresponding power output (W).

Test-retest reliability for the VO_2_peak protocol at the University of Oklahoma using twenty-one men, demonstrate reliable between-day testing with an intraclass correlation coefficient (ICC) of 0.975 (SEM 0.257 l·min^-1^) and a percent of coefficient of variation (%CV) of 5.18%.

### Total Work Done Cycling Test

Each subject performed a constant-load time to exhaustion (TTE) test on an electronically braked cycle ergometer, at a cadence of ~70 rpm. Participants performed a five minute warm-up at 50 W, followed by a cycle to exhaustion at their individual pre-determined workload, established at 110% of the maximum VO_2_peak workload (W). The subject's TTE was defined by the time (in seconds), that could be maintained without dropping below a cadence of 60 rpm. Total work done (TWD) was further calculated as the primary variable of interest, using the product of time (in seconds) and the power output (W), divided by 1,000, and presented in kilojoules (kJ).

The reliability statistics for TWD reflect a strong ICC of 0.713 (SEM 25.2 kJ) and a %CV of 3.80%.

### Training intervention and β-alanine supplementation

**Figure 1:**
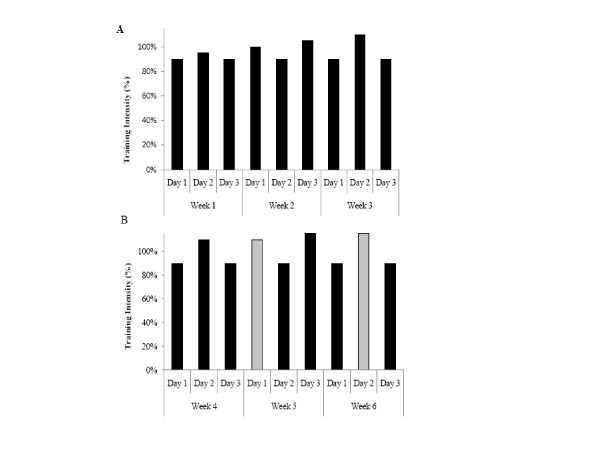
**Training protocol for the first and second three-week training phases, respectively**. Black represents five sets of the 2:1 training, while grey represents six sets of the same 2:1 protocol.

**Figure 2:**
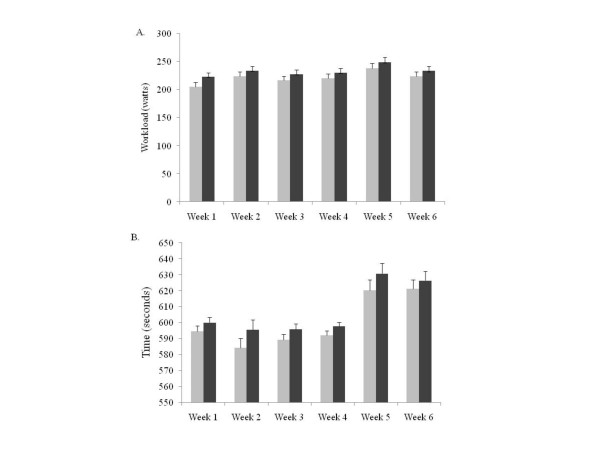
**2A. The average ± SD weekly training load (2A; watts) and training time (2B; seconds) between the BA (black) and PL (grey) treatment groups, across the six-week training protocol**.

Training was performed on an electronically braked cycle ergometer (Corval 400, Groningen, The Netherlands) to maintain testing specificity. Participants began the supervised training session within two to four days following testing. Following the baseline-testing and group randomization, subjects began the first of two, three-week training periods. Training followed a fractal periodized plan to allow for adequate progression and to prevent overtraining [32] and was completed three days per week. The training intensity began at 90% of the maximum power output (W) achieved during the baseline VO_2_peak test and progressed in an undulating manner, reaching a maximum of 115% by the end of the second, three-week training period. The first three-week period consisted of five sets of two-minute intervals with one-minute rest periods. The second three-week session followed a similar protocol, modifying the progression by increasing the repetitions from five to six, during weeks six and seven and still taking place on three days per week (Figure 1). A training log was completed for each training session. The total time (seconds) completed and workload (watts) was used to compute total training volume (kJ) (Figure 2).

In addition to training, during the first three-week period, the participants also supplemented with 6 g per day β-alanine (1.5 g β-alanine, 15 g dextrose per dose) or placebo (16.5 g dextrose per dose). Supplements were mixed with water in an orange flavored dextrose powder and were consumed four times throughout the day. On the three days that subjects visited the lab for training, they consumed two pre-mixed doses, one 30 minutes before, and one immediately after completion of the training session. The remaining two doses were taken that day, *ad libitum*. For the remaining four days of the week, participants were instructed to mix and consume the four doses (6 g per day) of their respective supplement, *ad libitum*. Throughout the second three-week training period, participants supplemented in a similar manner for on- and off-training days, for an additional 21 days, at a dose of 3 g per day, taken in two, 16.5 g doses (1.5 g β-alanine, 15 g dextrose). The participants in the placebo group consumed an isovolumetric flavored powder (16.5 g dextrose) identical in appearance and taste to the β-alanine. Participants were asked to record each dose on a designated dosing log for each day and they were asked to bring in the supplement packaging to allow investigators to monitor compliance.

### Determination of body composition

Body composition was assessed prior-to, mid-way, and following training and supplementing by using air displacement plethysmography (Bod Pod^®^). The subjects' weight (kg) and body volume were measured and used to determine percent body fat, fat mass (kg), and lean body mass (kg) using the revised formula of Brozek et al. [33].

### Statistical analysis

Separate two-way repeated measures ANOVAs (group [β-alanine vs. placebo] × time [pre- vs. mid- vs. post-supplementation]) were used to identify any group by time interactions. If a significant interaction occurred, the statistical model was decomposed by examining the simple main effects with separate one-way repeated measures ANOVAs for each group and one-way factorial ANOVAs for each time. An alpha of p ≤ 0.05 was used to determine statistical significance. All data are reported as mean ± standard deviation (SD).

## Results

Table 1 presents the mean and standard deviation values for VO_2_peak (l·min^-1^), VO_2TTE _(seconds), VT (watts) and TWD (kJ) for both treatment groups at pre-, mid- and post-testing.

**Table 1:** Mean ± SD values for VO_2_peak (l·min-1), VO_2TTE _(s), VT (W) and TWD (kJ) at pre-, mid-, and post-testing.

		Maximal Oxygen Consumption (l·min-1)		Time to Exhaustion (s)		Ventilatory Threshold (W)		Total Work Done (kJ)	
					
		β-alanine	Placebo	β-alanine	Placebo	β-alanine	Placebo	β-alanine	Placebo
Pre-test	Mean	3.28	3.25	1168.2	1128.7	140.3	127.3	58.4	55.7
	SD	0.57	0.63	163.6	166.9	35.5	42.6	19.2	13.8
Mid-test	Mean	3.52*	3.56*	1304.9*	1258.7*	154.2	140.3	89.0*	83.3*
	SD	0.49	0.56	153.7	204.5	36.6	52.3	30.1	25.7
Post-test	Mean	3.67†	3.66	1386.7†	1299.6	172.2	188.9†	131.3†	102.0†
	SD	0.58	0.55	234.9	164.9	65.2	58.3	81.7	36.7

*indicates a significant difference from pre- to mid-testing (p < 0.05)

†indicates a significant improvement from mid- to post-testing (p < 0.05)

### VO_2_peak, VO_2TTE_, VT during GXT

Significant main effects for time resulted for maximal oxygen consumption (VO_2_peak), time to exhaustion (VO_2TTE_) and ventilatory threshold (VT) determined during the graded exercise (p < 0.001). There were significant improvements in VO_2_peak after three weeks of training and supplementing across both treatment groups (p < 0.001; ES: 0.977). While there were no significant difference for the improvements in VO_2_peak at any time point between groups, only the BA group demonstrated significant improvements from mid- to post-training and supplementing (p = 0.010) with no significant change from mid- to post- for the PL group (p = 0.118). Similar results for VO_2TTE _were also revealed with both groups demonstrating significant improvements from pre- to mid-testing (p < 0.001; ES: 0.983), with no difference between groups. Significant changes from mid- to post-VO_2TTE _were only evident in the BA group (p = 0.043).

There were no significant differences among the improvements in VT between groups. Improvements from pre- to mid VT for both the PL and BA groups did not yield significance. However, the PL group was the only group to demonstrate significant improvements from mid- to post (p = 0.001).

### Time to exhaustion test-TWD

The improvements in TWD were significant across all time points, with no difference between groups (p > 0.05; ES: 0.898). While not significant, the delta values at both time points were greater for the BA group [pre-mid: 30.6 ± 19.9 sec; mid-post: 42.3 ± 72.1 sec] when compared to the PL group [pre-mid: 27.6 ± 22.1; mid-post: 18.6 ± 28.3].

### Body Composition

The physical characteristics of the subjects determined at mid-testing and after six-weeks of HIIT and supplementing are presented in Table 2. Body mass did not change significantly with supplementing or training. However, the determination of body composition with the use of air displacement plethysmography (Bod Pod^®^) revealed a significant improvement from pre- to mid-testing in lean body mass in only the BA group (p = 0.011; ES: 0.985) and no change in the PL group (p = 0.138). Furthermore, there were no significant changes in percent body fat (p = 0.287) or fat mass (p = 984) between treatment groups after three and six weeks of HIIT and supplementation.

**Table 2:** Mean ± SD values for body weight (kg), body fat (%), lean body mass (kg), and fat mass (kg) from pre-, mid-, and post-testing.

	**β-alanine (n = 18)**			**Placebo (n = 18)**		
	
	Pre-testing	Mid-testing	Post-testing	Pre-testing	Mid-testing	Post-testing
Weight (kg)	78.8 ± 12.8	80.1 ± 13.0	79.8 ± 12.4	78.5 ± 11.3	79.3 ± 12.3	79.8 ± 11.9
Body Fat (%)	13.7 ± 6.3	13.7 ± 6.4	13.7 ± 5.6	16.1 ± 7.5	15.9 ± 8.3	16.0 ± 7.9
Lean Body Mass (kg)	67.6 ± 8.9	68.6 ± 8.6*	68.4 ± 8.4	65.5 ± 8.1	66.1 ± 8.5	65.8 ± 8.4
Fat Mass (kg)	11.3 ± 6.5	11.5 ± 6.8	11.3 ± 6.0	13.0 ± 7.1	13.1 ± 8.0	13.0 ± 7.8

*indicates a significant difference from pre- to mid-testing. (p < 0.05).

### Dietary Analysis

There was no significant difference between groups for their supplement or training compliance rate, representing a 6.4 -3.2 g per day intake for the BA group, for the three and six weeks, respectively. Analyses of the dietary recalls demonstrated no significant differences in caloric intake (p > 0.05) between the BA (3120 ± 244 kcal) and placebo (2775 ± 209 kcal) groups. Furthermore, there were no differences in macronutrient daily intake, with both groups consuming 47% of their daily calories from carbohydrates, 34% from fat and 16% from protein.

### Training Volume

There was a significant main effect for time (p < 0.01) for both training volume (watts) and training time (seconds). However, there was no significant difference between groups for either volume (Figure 2A) or time (Figure 2B), at any time point (weeks 1–6). Although not significant, the BA group consistently trained at higher workloads and for longer time periods.

## Discussion

The current study is the first to examine the effects of concurrent high-intensity interval training (HIIT) and β-alanine supplementation on a series of physiological and performance variables. The primary findings support the use of HIIT as an advantageous training tool. Furthermore, the current study also proposes the use of β-alanine supplementation to enhance the benefits of HIIT, by possibly improving muscle buffer capacity after six weeks of training and supplementing. The maximal oxygen uptake and time to reach maximum oxygen consumption (VO_2_peak, VO_2TTE_) and total work done (TWD) increased significantly in both training groups (β-alanine and placebo) over a six week HIIT protocol (Table 1). However, β-alanine supplementation appeared to have a greater influence on VO_2_peak and VO_2TTE_, resulting in a significant (p < 0.05) increase during the second three weeks of training, while no change occurred in placebo group. In addition, TWD significantly (p < 0.05) increased during the last three weeks by 32% and 18% for the β-alanine and Placebo groups, respectively. Improvements in VT were also reported for both training groups, however the placebo group demonstrated significant improvements during the last three week training phase (Table 1). Lastly, the present study also identified a significant change in lean body mass for the β-alanine supplementing group after three weeks, with no change in the placebo group.

### Enhanced VO_2_peak, VO_2TTE_, and VT after training

A series of HIIT interventions have suggested that interval exercise (> 80% VO_2_max) elicits greater gains in aerobic capacity than moderate-intensity exercise [34-36]. Consequently, the improvements reported in cardiorespiratory fitness in the current study were similar to most studies that have employed short-term (2–9 weeks) endurance interval training programs in untrained and recreationally active individuals [25,29,34,37-40]. Specifically, the average reported increases in VO_2_peak have ranged from 6–20% in male and female populations. Although the training regimens utilized have varied slightly, all supporting studies applied a similar protocol. The use of a 1:1 [37,38,40] and a 2:1 [29,34,39] work-to- rest design (1–4 minutes) have been the most effective for promoting an increase in aerobic capacity. Our data supports previous literature, suggesting a 7–10% increase in VO_2_peak during the first three week training phase and a 3–4.5% increase following the second three week session. While both groups significantly improved in VO_2_peak and VO_2TTE _from pre- to mid-testing, only the β-alanine group demonstrated significant improvements from mid- to post-testing (Table 1).

The use of high-intensity exercise as a training modality has been shown to stimulate acute and chronic physiological adaptations (cardiovascular, metabolic, respiratory and neural), which ultimately lead to improved performance [34,37,41]. The increases in VO_2_peak, VO_2TTE_, and VT reported in the current study are in line with other studies, which have suggested that the improvements in aerobic performance are attributable to a reduction in anaerobic ATP production, resulting from an increased contribution of aerobic energy production at higher intensity workloads [42,43]. The greater reliance on aerobic metabolism for energy has been further linked to an up-regulation of various glycolytic enzymes (phosphofructokinase, hexokinase, citrate synthetase, and sodium potassium ATPase) [42,44-47], as well as with increased mitochondrial density and improved blood flow due to increased capillarization [44,45]. These improvements, in combination with an enhanced ability to buffer H^+^, may provide some explanation into the greater improvements in the second three-week training phase, in the BA group only. Although blood pH levels were not measured directly, support from training volume (Figure 2A) and training time (Figure 2B), demonstrate that participants supplementing with β-alanine engaged in longer, more intense training sessions, possibly leading to greater adaptations.

### Improvements in TWD

In addition to augmenting VO_2_peak, VO_2TTE _and VT, the HIIT program utilized in the current study demonstrated significant improvements in TWD (Table 1). Interestingly, the increases in total work performed in the current study were greater than in previously reported improvements in TWD following HIIT alone [48-50], with both groups demonstrating a 50–53% improvement during the first three weeks of training and the β-alanine group showing a 32% increase compared to the 18% increase in the placebo group, after the second three-week training phase. In support, Kim et al. [21] demonstrated significantly greater increases in TWD in highly trained cyclists after a 12-week β-alanine supplementation and endurance training program, compared to training only. In addition, Hill et al. [6] also demonstrated significant improvements in TWD (13%) on a cycle ergometer following four weeks of β-alanine supplementation, without training. While the data appear to support the use of β-alanine supplementation to augment TWD, with and without training, the previously mentioned studies utilized highly trained participants, compared to an un-trained population in the current study.

Scientists have suggested the use of β-alanine may enhance training adaptations [6,18,23], by increasing ability to train at a higher intensity without fatigue. Recently Harris et al. [18] and Hill et al. [6] have posited that increasing skeletal muscle carnosine concentration with β-alanine supplementation may improve the ability to stabilize the intramuscular pH during intense exercise by buffering accumulating H^+^. Offsetting the indirect effect of proton accumulation on contractile function with the use of β-alanine, has been shown to be effective in delaying neuromuscular fatigue, improving VT and time to exhaustion in both trained and untrained individuals [6,21,23,24]. Furthermore, Kim et al. [21] reported a significant increase in VT after 12 weeks of endurance and resistance training while supplementing β-alanine in highly trained cyclists. However, our results demonstrated no added benefit of combining β-alanine supplementation and HIIT to elicit increases in VT, greater than training alone. The differences in training status (elite vs. recreationally trained) may have resulted in the conflicting results between the current study and Kim and colleagues. Additional research examining the effects of concurrent β-alanine supplementation and HIIT in trained versus untrained men and women would provide additional insight toward the current findings.

### Augmented Lean Body Mass

Interestingly, the improvements in performance over the six-weeks of training also demonstrated concomitant gains in lean body mass in the β-alanine group only. Recent evidence suggests that intense exercise may elicit intramuscular acidosis, potentially augmenting protein degradation [51], inhibiting protein synthesis [52] and thus hindering training adaptations. Another theory posited suggests that β-alanine supplementation may have allowed for greater training volume thus providing a greater stimulus, resulting in significant gains in lean body mass, as observed in the current study. In support, Hoffman et al. [53,54] reported significantly higher training volume for athletes consuming β-alanine during resistance training sessions, which they hypothesized lead to significant increases in lean body mass. In short, minimizing the acidic response from HITT, and/or increasing training volume with β-alanine supplementation, may help to increase lean body mass and lead to improvements in performance.

## Conclusion

Our findings support the use of HIIT as an effective training stimulus for improving aerobic performance, in as little as three weeks. The use of β-alanine supplementation, in combination with HIIT, appeared to result in greater changes in VO_2_peak and VO_2TTE_, during the second three weeks of training, while no significant change occurred in placebo group. In addition, TWD significantly (p < 0.05) increased during the last three weeks by 32% and 18% for the β-alanine and Placebo groups, respectively. While more research is needed, the current study suggests that in untrained young men, the use of β-alanine supplementation may enhance the benefits of HIIT and augment endurance performance.

## Competing interests

The authors declare that they have no competing interests.

## Authors' contributions

All authors contributed equally to this work. All authors have read and approved the final manuscript.
